# Autophagy mediates epithelial cancer chemoresistance by reducing p62/SQSTM1 accumulation

**DOI:** 10.1371/journal.pone.0201621

**Published:** 2018-08-01

**Authors:** R. Alessia Battista, Massimo Resnati, Cecilia Facchi, Elena Ruggieri, Floriana Cremasco, Francesca Paradiso, Ugo Orfanelli, Leone Giordano, Mario Bussi, Simone Cenci, Enrico Milan

**Affiliations:** 1 Age Related Diseases Unit, Division of Genetics and Cell Biology, San Raffaele Scientific Institute, Milano, Italy; 2 Università Vita-Salute San Raffaele, Milano, Italy; 3 Department of Otorhinolaryngology, San Raffaele Scientific Institute, Milano, Italy; Niigata Daigaku, JAPAN

## Abstract

To cope with intrinsic and environmental stress, cancer cells rely on adaptive pathways more than non-transformed counterparts. Such non-oncogene addiction offers new therapeutic targets and strategies to overcome chemoresistance. In an attempt to study the role of adaptive pathways in acquired drug resistance in carcinoma cells, we devised a model of *in vitro* conditioning to three standard chemotherapeutic agents, cisplatin, 5-fluorouracil, and docetaxel, from the epithelial cancer cell line, HEp-2, and investigated the mechanisms underlying reduced drug sensitivity. We found that triple-resistant cells suffered from higher levels of oxidative stress, and showed heightened anti-stress responses, including the antioxidant Nrf2 pathway and autophagy, a conserved pleiotropic homeostatic strategy, mediating the clearance of aggregates marked by the adapter p62/SQSTM1. As a result, re-administration of chemotherapeutic agents failed to induce further accumulation of reactive oxygen species and p62. Moreover, autophagy proved responsible for chemoresistance through the avoidance of p62 accumulation into toxic protein aggregates. Indeed, p62 ablation was sufficient to confer resistance in parental cells, and genetic and pharmacological autophagic inhibition restored drug sensitivity in resistant cells in a p62-dependent manner. Finally, exogenous expression of mutant p62 lacking the ubiquitin- and LC3-binding domains, required for autophagic engulfment, increased chemosensitivity in TDR HEp-2 cells. Altogether, these findings offer a cellular system to investigate the bases of acquired chemoresistance of epithelial cancers and encourage challenging the prognostic and antineoplastic therapeutic potential of p62 toxicity.

## Introduction

Tumorigenesis is a multistep, mutagenic process whereby transformed cells acquire a set of phenotypic “hallmarks” that allow them to survive, proliferate and metastasize [1]. Cancer transformation occurs through genomic mutations in diverse oncogenes and oncosuppressor genes, combined with a large number of low-frequency tumor-specific genetic changes, generating a great complexity in cancer pathobiology. However, although necessary for cancer development, genetic mutations do not account for the entire malignant phenotype. Indeed, striving to survive in a challenging environment, characterized, among other elements, by hypoxia, nutrient starvation and therapy-induced toxicity, malignant cells have to cope with different stresses, such as proteotoxic, mitotic, metabolic and oxidative stress, and thus rely on diverse adaptive pathways more than normal counterparts [2]. Such *non-oncogene addiction* of cancer offers a previously unimaginable framework of therapeutic opportunities, especially in those tumors characterized by narrow therapeutic window and poor prognosis due to chemoresistance. This holds particular promise for those cancers that failed to show substantial increases of patient survival rates in the last decades (e.g., head and neck cancers). Based on this rationale, in this study we aimed to dissect the role of cellular stress response pathways, and in particular those involved in protein homeostasis (proteostasis), in chemotherapy sensitivity.

Cellular proteostasis is ensured by multiple pathways regulating the synthesis, folding, localization and degradation of proteins, including the heat-shock response, the unfolded protein response (UPR), and the two principal proteocatabolic pathways: the ubiquitin-proteasome system (UPS) and macroautophagy (conventionally referred to as autophagy) [3]. Autophagy is a conserved recycling strategy consisting in the engulfment of substrates in double membrane vesicles, called autophagosomes, which eventually fuse to lysosomes for content digestion. Cargo selection is achieved through autophagic adapters, the prototype of which is SQSTM1/p62, that crosslink LC3-decorated autophagosomal membranes with ubiquitinated targets destined to degradation [4–5]. Moreover, p62 controls additional adaptive responses, like the Nrf2 antioxidant pathway [6]. Indeed, Nrf2 is a transcription factor constitutively degraded by the UPS, through the inhibitory interaction with the redox sensor Keap1. Under stress conditions that lead to p62 accumulation, binding of this autophagic adapter with Keap1 stabilizes Nrf2, inducing its translocation to the nucleus to transactivate a broad spectrum of antioxidant response element (ARE)-containing genes encoding, among others, canonical anti-oxidant proteins such as HMOX1, NQO1 and GCLM [7–10]. Dissecting the role of these fundamental cellular stress response pathways in mediating cancer chemoresistance might open new perspectives to increase therapeutic efficacy. To explore this issue, we generated new models of chemoresistance adopting the carcinoma cell line HEp-2 and investigated the role of resistance-associated adaptive responses *in vitro* by biochemical profiling and genetic and pharmacological dissection.

## Materials and methods

### Cell line

Human epithelial HeLa-derived HEp-2 cell line (ATCC^®^ CCL-23^™^), purchased from ATCC (American Type Culture Collection), was cultured in Dulbecco’s modified Eagle’s medium (DMEM, Gibco-Life Technologies, Carlsbad, CA) supplemented with 10% fetal bovine serum (Euroclone), L-glutamine (2 mM; Gibco-Life Technologies, Carlsbad, CA), penicillin (100 U/ml; Lonza, Basel, Switzerland), streptomycin (100 μg/ml; Lonza, Basel Switzerland) and sodium pyruvate (1 mM; Sigma-Aldrich, Saint Louis, MO). The cells were grown as monolayer cultures and maintained in a humidified atmosphere of 5% CO2 at 37 °C. HEp-2 cells were treated at the indicated doses with Cisplatin [cis"diammineplatinum (II) dichloride], Docetaxel, 5-FU [5-fluorouracil] and Bafilomycin-A1 (Sigma-Aldrich, Saint Louis, MO).

### Drug sensitivity assay

Parental and resistant HEp-2 were plated in duplicate at a concentration of 1x10^3 cells/well in 96-well plates. The cells were incubated overnight in humidified air with 5% CO2 at 37°C. They were subsequently treated with serial dilutions of drugs for 24 h, followed by further culture in drug-free medium for two days. In Bafilomycin-A1 treated MTT assay the drug (10 nM, Sigma Aldrich, Saint Louis, MO) was added together with chemotherapeutic drugs for 24 h. MTT assay was then performed, according to the manufacturer’s instructions. Briefly, 20 μl MTT (5 mg/ml; Sigma-Aldrich, Saint Louis, MO) was added to the culture medium and the cells incubated for 4 h at 37°C. The formazan crystals were dissolved by adding 120 μl DMSO per well, previous culture medium removal, and the plate was read at 570 nm, as the reference wavelength, using a microplate reader (Model 680; Bio-Rad Laboratories Inc, Hercules, CA). Not treated cells were used as control, wells without cells were used as blank, whose mean optical density (OD) was subtracted to sample OD to normalize. The percentage of viable cells was calculated using the formula: Mean optical density (OD) treated cells / mean OD control cells x100. IC50 values in each case were calculated using Prism software, version 4.0 (GraphPad). The resistance index (RI) was calculated by the ratio of the IC50 of resistant cell lines over the parental cell lines.

### Lentiviral transduction

Plasmid constructs expressing anti-p62 and anti-ATG7, and control shRNAs were obtained with Mission shRNAs pLKO.1-puro homemade modified to express GFP (nontarget shRNA: SHC002; sh*p62*: TRCN0000007234; shAtg7: TRCN0000007584; Sigma-Aldrich, Saint Louis, MO). Human FLAG-p62 cDNA was purchased from Genscript (Piscataway, NJ) and cloned in a plasmid with a bidirectional human PGK promoter co-expressing p62 and truncated human LNGFR. FLAG-G263X mutant p62 was generated by specific PCR from the original wild-type cDNA (Genscript). Lentiviral vectors were packaged with Sigma-Aldrich Mission shRNAs or FLAG-p62 cDNAs, pMD2-VSV-G, pMDLg/pRRE and pRSV-Rev plasmids in HEK 293T. HEp-2 and TDR HEp-2 were infected with concentrated viruses and polybrene (8 μg/ml). Flow cytometry was performed 3 days after lentiviral infection to determine the GFP-positive proportion of cells.

sh*p62* sequence: CCGGCGAGGAATTGACAATGGCCATCTCGAGATGGCCATTGTCAATTCCTCGTTTTT
shAtg7 sequence: CCGGGCCTGCTGAGGAGCTCTCCATCTCGAGATGGAGAGCTCCTCAGCAGGCTTTTT

### ROS quantification

HEp-2 cells were treated at the indicated doses of CT agents then collected and stained with CM-H2DCFDA (Life Technologies, Carlsbad, CA) 10μM for 30 minutes in PBS. Cells were washed and analyzed at Accuri C6 Flow Cytometer.

### Western blot

Total cellular extracts were obtained by lysis in 150 mM NaCl, 10 mM Tris-HCl (pH 7.5) and 1% SDS supplemented with protease inhibitors. Genomic DNA was sheared by 30’ sonication, then proteins were quantified by Bradford assay. Protein in a range of 15–40 μg were resolved by 10%, 12% or 15% SDS-PAGE and blotted on nitrocellulose membrane (Mini-PROTEAN Tetra Cell, BioRad, Hercules CA). Membranes were blocked in 5% milk in 0.1% TBS-Tween, incubated with primary and secondary antibodies, thoroughly washed with 0.1% TBS-Tween. Proteins were revealed by ECL at ChemiDoc-it (UVP, Upland CA) for HRP-conjugated secondary Ab or at FLA9000 (FujiFilm, Minato, Japan) for Alexa Fluor conjugated secondary antibodies (Life Technologies, Carlsbad, CA). Band densitometric analysis was performed in ImageJ free software (http://rsbweb.nih.gov/ij/). Antibodies used for blotting were: guinea pig anti-p62 C terminal pAb (1:1000 dilution; GP62-C, ProGen, Brisbane, Australia), rabbit anti-p62 (1:1000; P0067; Sigma-Aldrich, Saint Louis, MO) rabbit anti-LC3 pAb (1:500, NB100-2331; Novus Biologicals, Littleton, CO), mouse anti-actin (1:4000; Clone AC-15; Sigma-Aldrich, Saint Louis, MO), rabbit anti-Nrf2 (1:700; Clone H300; Santa Cruz Biotechonology Inc, Dallas, TX), mouse anti-PSMA2 (PW8105 Enzo Life Sciences, Farmingdale, NY), rabbit anti-PSMB5 (PW8895 Enzo Life Sciences), rabbit anti-PDI (kind gift of Ineke Braakman, Utrecht, NL), anti rabbit-NBR1 (9891, Cell Signaling, Leiden, Netherlands) goat anti-BiP (sc-1051 Santa Cruz Biotechonology Inc, Dallas, TX).

### Quantitative RT-PCR

For qRT-PCR, RNA was extracted from 1x10^6 cells with Trizol (Life Technologies, Carlsbad, CA), 1000 ng RNA retrotranscribed with ImProm-II Reverse Transcriptase (Promega, Madison, WI), and cDNA corresponding to 2–5 ng of original RNA used as template in qRT-PCR reactions. qRT-PCR was performed in 10μl mix with 5μl SYBR green I master mix (Roche, Basel, Switzerland) on Roche LightCycler480 and with 1 μl 5 μM primers. Data were analyzed on Roche LC480 software with Advance Relative Quantification. Human H3 expression was used as normalizer in the analyses. Primers used were the following:

GCLM: forward (FW), TCAGTCCTTGGAGTTGCACA; reverse (REV), ACACAGCAGGAGGCAAGATT;
H3: FW, GTGAAGAAACCTCATCGTTACAGGCCTGGT; REV, CTGCAAAGCACCGATAGCTGCGCTCTGGAA;
HMOX1: FW, GCAACCCGACAGCATGCCCC; REV, CAGCGGGGCCCGTACCAGAA;
NQO1: FW, GGAGAGTTTGCTTACACTTACGC; REV, AGTGGTGATGGAAAGCACTGCCTTC;
PGD: FW, TAGGGACACCACAAGACGGT; REV, CCCACTTTTGCAGCAATGCC;
ME1: FW, GCAGCTCTTCGAATAACCAAG; REV, CAATCAGGTGTGCAATCCCTA;
TKT: FW, GGATGACCAGGTGACCGTTA; REV, CGCGGATGTTGATCTTTTCT;
SLC7A11: FW, CCATGAACGGTGGTGTGTT; REV, GACCCTCTCGAGACGCAAC;
p62: FW, GGGGCGCCCTGAGGAACAGA; REV, CCTGGTGAGCCAGCCGCCTT;
NBR1: FW, GCGAGCTGAGAAGAAACAACG; REV, GAAGGTGAGTCCCATCAGGC;
ATG7: FW, TGGAACAAGCAGCAAATGAG; REV, AGACAGAGGGCAGGATAGCA;
ATG5: FW, AAGCTGTTTCGTCCT; REV, AGCCACTGCAGAGGT;
ATG6/BECN1: FW, AGGATGATGTCCACAGAAAGTGC; REV, AGTGACCTTCAGTCTTCGGCTG;
ATG1/ULK1: FW, TCGAGTTCTCCCGCAAGG; REV, CGTCTGAGACTTGGCGAGGT;
Hsp60: FW: TGCTCACCGTAAGCCTTTGG; REV, AAACCCTGGAGCCTTGACTG;
CHOP: FW, GAGCTGGAACCTGAGGAGAGA; REV, GCAGTTGGATCAGTCTGCTT;
sXBP1: FW, CCGCAGCAGGTGCAGG; REV, GAGTCAATACCGCCAGAATCCA;
tXBP1: FW, GCAAGCGACAGCGCCT; REV, TTTTCAGTTTCCTCCTCAGCG;
PSMB5: FW, TCAGTGATGGTCTGAGCCTG; REV, CCTTCTTCACCGTCTGGGAG.

### Fluorescence microscopy analysis

HEp-2 cells were seeded on slides, fixed with 4% paraformaldehyde, and permeabilized with PBS 0.1% Triton X-100. Cells were stained with guinea pig SQSTM1 antisera (1:200), C-terminal (Progen, GP62-C, Brisbane, Australia), rinsed in PBS, and stained with Alexa Fluor 488 goat anti-guinea pig IgG (1:500, Life Technologies, Carlsbad, CA) and Hoechst 33,342 (Life Technologies, Carlsbad, CA). Images were acquired with Leica TCS SP8 confocal microscope with a 63× objective (oil) with a numerical aperture of 1.4.

### mCherry-EGFP-LC3B reporter

The plasmid expressing mCherry-EGFP-LC3B as originally described [11] was transfected in HEp-2 cells using Lipofectamine 3000 (Invitrogen, Carlsbad, CA) following manufacturer’s instructions. After 72h mCherry-EGFP-LC3B was analyzed by immunofluorescence at Leica TCS SP8 confocal microscope with a 63× objective (oil) with a numerical aperture of 1.4 or by cytofluorimetry with CytoFLEX Flow Cytometer (Beckman Coulter, Brea, CA).

### Statistical and data analysis

Graphs and data analysis were performed in Prism v6.0 (GraphPad).

Drug sensitivity assays were analyzed by two-way ANOVA coupled with Bonferroni post-hoc tests for simple main effects.

Gene and protein expression analyses by RT-PCR and immunoblot (fold changes, HEp-2 vs TDR) were carried out by non-parametric Wilcoxon signed-rank test.

ROS levels, LC3 fluxes and RT-PCR with multiple groups were analyzed by one-way ANOVA coupled with Bonferroni post-hoc test between the groups.

## Results

### Generation of a chemoresistant cancer cell line model

**Fig 1:**
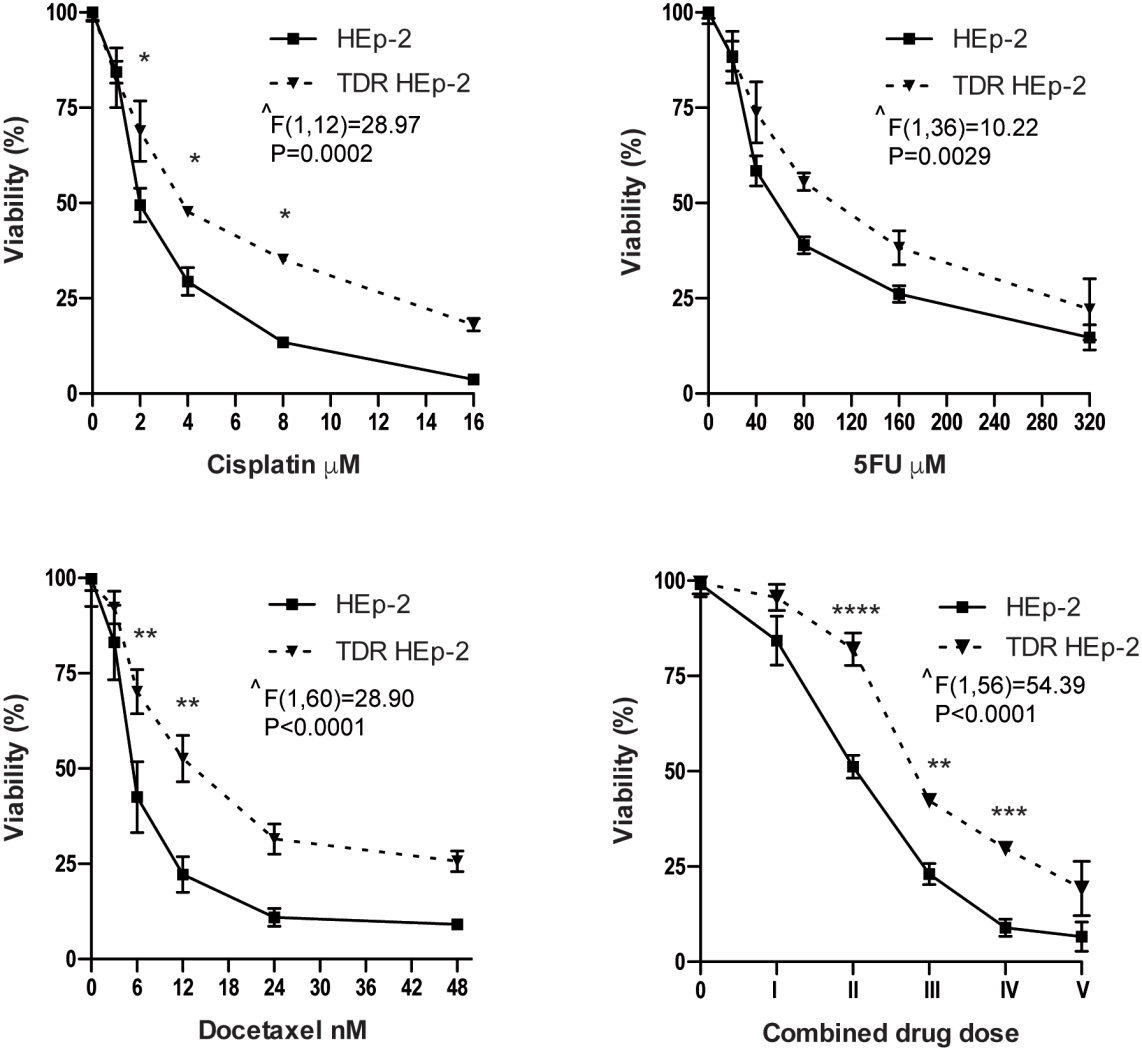
Sensitivity of parental and TDR HEp-2 to 24 h treatment with the indicated doses of cisplatin, 5-FU, docetaxel and their combination, as assessed by MTT assay. Panels show dose-response viability curves to the indicated treatments. The right bottom panel shows the viability of TDR HEp-2 cells exposed to five combined drug doses containing progressively doubling concentrations of cisplatin (μM), 5-FU (μM) and docetaxel (nM), respectively: **I**, 1, 20, 3; **II**, 2, 40, 6; **III**, 4, 80, 12; **IV**, 8, 160, 24; **V**, 16, 320, 48. Mean ± standard error of the mean (SEM). Two-way ANOVA (resistance x treatment) with Bonferroni post-hoc test, *p < 0.05; ** *p* < 0.01; *** *p* < 0.001; **** *p* < 0.0001; n ≧ 3); **^** main effect of resistance.

To generate a chemoresistant epithelial carcinoma model, we cultured HEp-2 cells in progressively increasing concentrations of three widely used chemotherapeutic agents known to exert direct cytotoxicity against epithelial malignancies: cisplatin, 5-fluorouracil (5-FU), and docetaxel [12]. We designed a conditioning protocol using three incremental doses of each drug (S1 Table). Briefly, HEp-2 cells were treated with the first dose of each drug, combined or separate, for 24 h, followed by further culture in drug-free medium for 7–10 days, collected, and the process repeated with the second and then the third dose set. In this way, we selected a putatively triple drug resistant cell population (TDR HEp-2), as well as three cell populations conditioned by each single agent. We then verified drug de-sensitization by challenging the cell lines obtained with increasing doses of each chemotherapeutic agent, single or combined, and assessing viability by MTT assay. TDR HEp-2 cells were significantly more resistant than parental cells to cisplatin, 5FU, docetaxel, or their combination (Fig 1). Exposure to increasing doses of single agents induced more modest de-sensitization, which appeared consistent and significant only for cisplatin and docetaxel, as compared to parental HEp-2 cells (S1 Fig).

A resistance index (RI) was then calculated as the ratio of the half maximal inhibitory concentration (IC50) of drug-conditioned cell lines over parental HEp-2 cells (Table 1).

**Table 1:** Half maximal inhibitory concentration (IC50) and resistance index (RI) of parental and drug-conditioned HEp-2 cells.

Cell lines	Drug	IC50	RI
parental HEp-2	cisplatin (μM)	2.11	1
	5-FU (μM)	53.92	1
	docetaxel (nM)	3.22	1
cisplatin-conditioned HEp-2	cisplatin (μM)	2.63	1.25
5-FU-conditioned HEp-2	5-FU (μM)	76.40	1.42
docetaxel-conditioned HEp-2	docetaxel (nM)	6.66	2.07
TDR HEp-2	cisplatin (μM)	3.82	1.81
	5-FU (μM)	82.55	1.53
	docetaxel (nM)	13.44	4.17

Single agent-conditioned HEp-2 cells showed an IC50 increased by 25, 42, and 107%, when treated with cisplatin, 5-FU and docetaxel, respectively, while the increase was 81%, 53% and over 400% in TDR HEp-2 cells. Notably, the IC50 of each single agent was invariably higher in TDR than single agent-conditioned HEp-2 cells, in line with the existence of mechanisms of cross-resistance. Accordingly, HEp-2 cells exposed to increasing doses of cisplatin and 5-FU developed identical resistance to both treatments, but none to docetaxel, whereas the sensitivity to cisplatin and 5-FU of cells exposed to increasing doses of docetaxel was comparable to that of parental cells (S2 Fig). Indeed, cisplatin and 5-FU act through similar DNA-interfering mechanisms, which are distinct from those targeted by docetaxel, *i*.*e*., microtubule dynamics.

### Higher proteostatic and anti-oxidant activity in TDR HEp-2 cells

**Fig 2:**
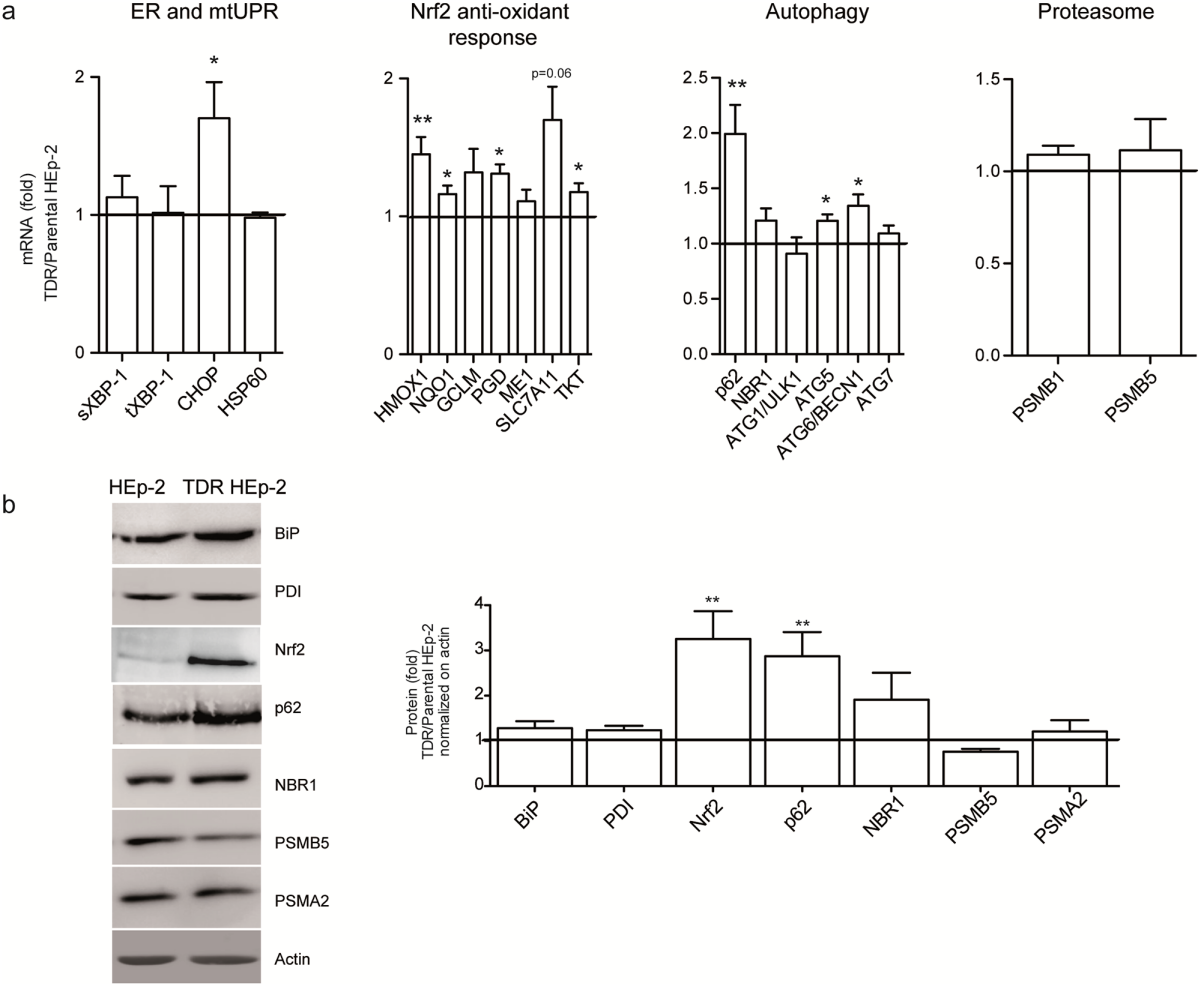
Increased p62 and Nrf2 expression in TDR HEp-2 cells. (**a**) mRNA abundance of the indicated genes representative of the canonical (*i*.*e*., ER stress-induced) UPR, Nrf2-dependent anti-oxidant response, autophagic factors, proteasome subunits, and the mitochondrial UPR (mtUPR), were assessed by Sybr Green qRT-PCR in parental and TDR HEp-2 cells and normalized to histone H3 levels (fold change TDR/HEp-2 ± SEM, Wilcoxon signed-rank test, *p < 0.05; ** *p* < 0.01; *** *p* < 0.001, n ≧ 5 independent experiments). (**b**) Immunoblot analysis of the indicated proteins representative of the pathways explored in (a). Left: one representative gel; right: quantification of minimum 3 independent experiments (fold change TDR/HEp-2 ± SEM, Wilcoxon signed-rank test, ** *p* < 0.01).

We then investigated the biological bases of chemoresistance in triple drug-conditioned HEp-2 cells. Quantitative RT-PCR (qRT-PCR) of transcripts involved in antioxidant and proteostatic pathways revealed increased expression of a number of genes. These included the autophagic genes ATG5, ATG6/BECN1, the prototypical autophagic receptor, p62/SQSTM1 and of the canonical Nrf2 targets (namely HMOX1, NQO1, TKT, PGD and SLC7A11) in TDR HEp-2 as compared to parental cells. Within the endoplasmic reticulum (ER) unfolded protein response (UPR), we found CHOP mRNA significantly higher in TDR cells, whereas spliced and total XBP-1 as well, as the paradigmatic mitochondrial UPR gene HSP60, were not differentially expressed. Moreover, no significant differences were observed in the expression of two representative β peptidase proteasomal subunits (Fig 2a). Western blot analysis confirmed the upregulation of p62 and Nrf2 proteins in TDR HEp-2 cells (Fig 2b). Increased expression of p62 and Nrf2 targets was also demonstrated in single agent-conditioned HEp-2 cells (S3 Fig).

**Fig 3:**
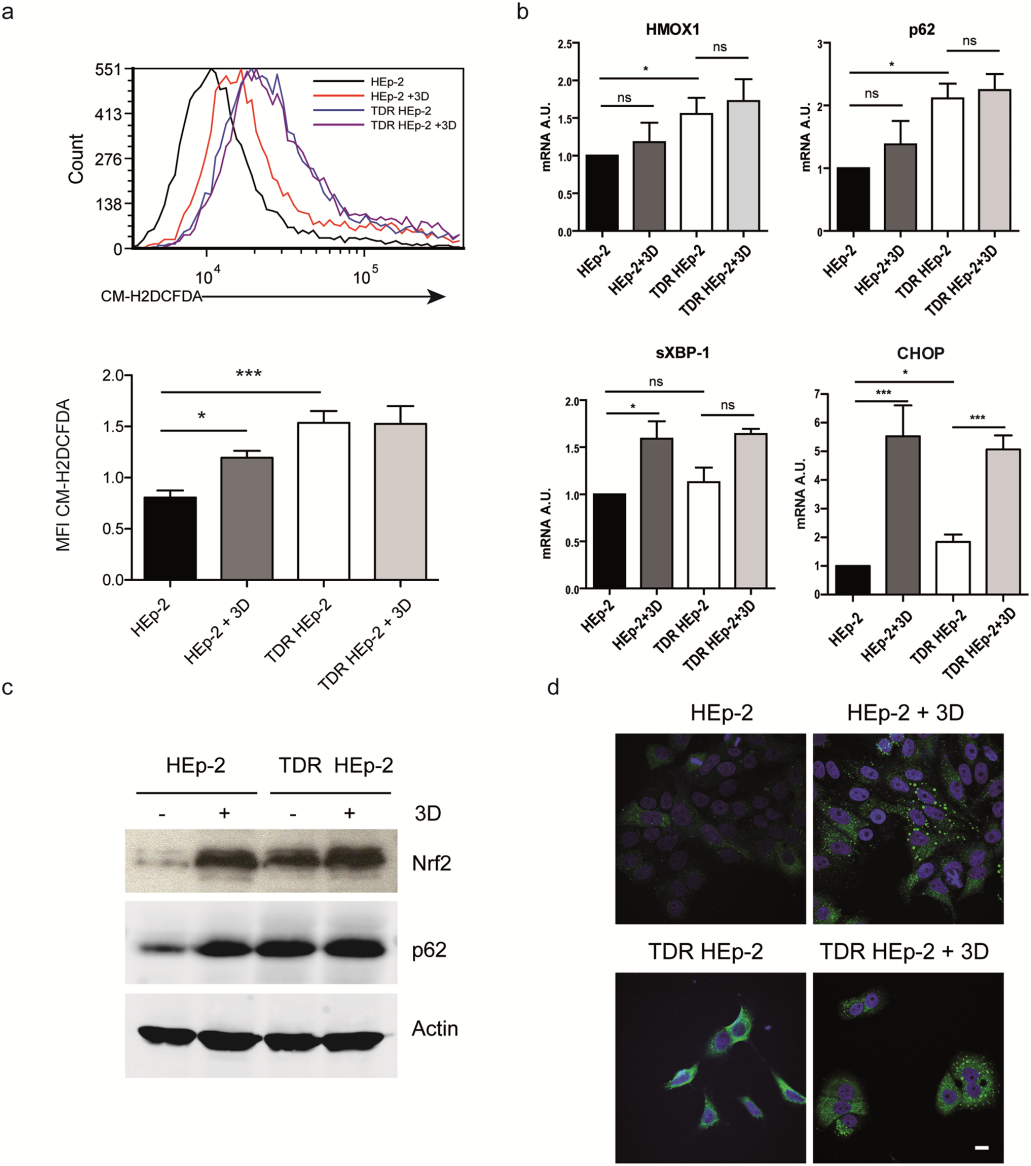
Higher anti-oxidant Nrf2 activity and p62 expression in TDR HEp-2 cells. **a**) Cytofluorimetric assessment of ROS content upon CM-H2DCFDA staining in parental and TDR HEp-2 cells treated with cisplatin 4 μM + 5-FU 80 μM + docetaxel 12 nM (three drugs, 3D) for 24 h. Bottom: quantitative representation of CM-H2DCFDA median fluorescence intensity (MFI) in each sample (one-way ANOVA with Bonferroni post-hoc test, *p < 0.05; ** *p* < 0.01; *** *p* < 0.001, n = 3). (**b**) Transcript expression of the Nrf2 target, HMOX1 and of the autophagy receptor p62, by qRT-PCR in parental and TDR HEp-2 cells, untreated or treated as in (a). Induction of the UPR transcripts, sXBP-1 and CHOP served as controls of drug effectiveness (mean ± SEM, one-way ANOVA with Bonferroni post-hoc test, *p < 0.05; ** *p* < 0.01; *** *p* < 0.001). (**c**) Immunoblot analysis of Nrf2 and p62 protein expression in parental and TDR HEp-2 cells treated as above. (**d**) Immunofluorescence analysis of p62 expression and intracellular distribution in parental and TDR HEp-2 cells treated as above. Scale bar: 10 μm.

We then aimed to define the adaptive relevance of the observed changes. Using a specific dye for reactive oxygen species (ROS), we found that TDR HEp-2 cells suffered from significantly higher basal oxidative stress than parental counterparts (Fig 3a). However, attesting to successful adaptation, acute re-administration of the highest conditioning dose of the combined drugs failed to further accumulate ROS in TDR HEp-2 cells, while it induced oxidative stress in parental HEp-2 cells (Fig 3a). Resistance to oxidative stress in TDR HEp-2 cells correlated with higher basal Nrf2 activity, as revealed by higher HMOX1 transcript expression (Fig 3b) and higher Nrf2 protein abundance (Fig 3c) in untreated TDR HEp-2 cells as compared to parental counterparts. Moreover, in parental HEp-2 cells, combined drug administration induced remarkable accumulation of p62 protein, in spite of insignificantly increased transcript levels (Fig 3b and 3c), hinting at p62 protein stabilization, possibly due to aggregation. Indeed, immunofluorescence analysis showed remarkable accumulation of p62 in large aggregates in parental HEp-2 cells upon combined drug treatment (Fig 3d). In contrast, TDR HEp-2 cells showed higher basal expression of p62 with a diffuse pattern (Fig 3c and 3d). Attesting to a causal role of p62 in activating Nrf2, the knockdown of p62 in TDR HEp-2 reduced Nrf2 protein abundance and the expression of Nrf2 target genes (S4 Fig). Taken together, the data show that increased drug resistance in TDR HEp-2 cells is associated with increased antioxidant Nrf2 activity and improved proteostatic capacity.

### Autophagy mediates chemoresistance in HEp-2 cells through clearance of aggregated p62

**Fig 4:**
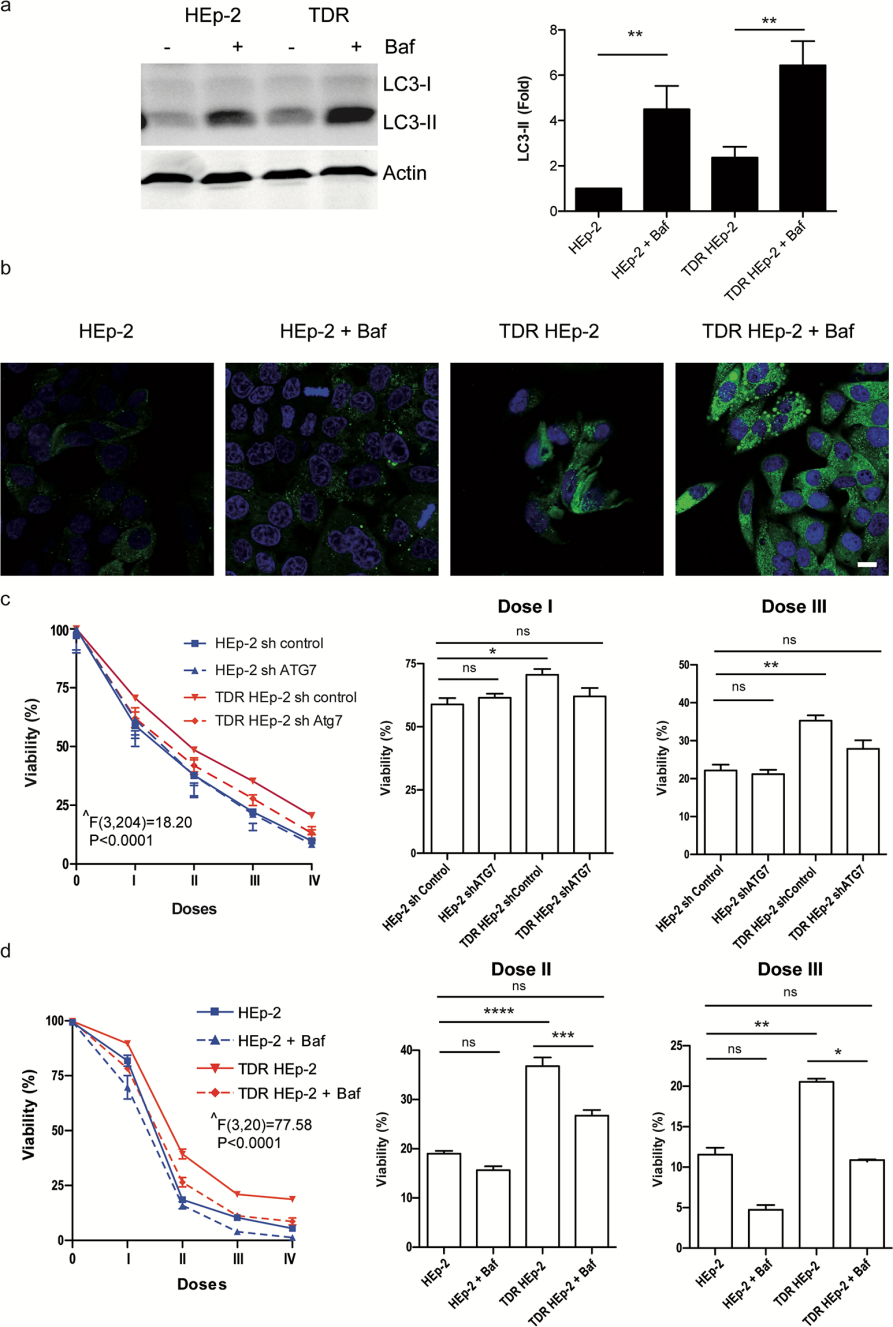
Autophagy confers chemoresistance in HEp-2 cells. (**a**) Immunoblot analysis of autophagic flux in parental and TDR HEp-2 cells, as estimated by the accumulation of LC3-II in the presence of 10 nM bafilomycin-A1 (Baf) for 16 h. Left: one representative gel; right: quantification of 5 independent experiments (mean ± SEM, one-way ANOVA with Bonferroni post-hoc test, ** *p* < 0.01). (**b**) Immunofluorescence analysis of p62 distribution and autophagic clearance in parental and TDR HEp-2 cells treated with Baf as in (a). Scale bar: 10 μm. (**c-d**) Effect of genetic autophagic blockade (ATG7 silencing) (**c**) and pharmacologic distal autophagic inhibition (Baf) (**d**) on the toxic effect exerted by the treatment with four combinations of progressively doubling concentrations of cisplatin (μM), 5-FU (μM) and docetaxel (nM), respectively: **I**, 1, 20, 3; **II**, 2, 40, 6; **III**, 4, 80, 12; **IV**, 8, 160, 24; n = 10 (**c**); n = 4 (**d**). Left: dose-response viability curves by MTT assay; right: toxicity exerted at the indicated doses (mean ± SEM). Two-way ANOVA (group x treatment) with Bonferroni post-hoc test, *p < 0.05; ** *p* < 0.01; *** *p* < 0.001; **** *p* < 0.0001); **^** main effect of group.

Autophagy is a critical cytoprotective strategy affording, among other functions, degradation of protein aggregates and antioxidant defense [3–5]. The correlation between drug-induced p62 aggregation and chemosensitivity (Fig 3) led us to hypothesize a causal role for autophagy in chemoresistance of TDR HEp-2 cells. To test this hypothesis, we first quantified total autophagic flux by measuring the rate of lysosomal digestion of phosphatidylethanolamine-conjugated LC3 (LC3-II), a short-lived marker of autophagosome membranes. Accumulation of endogenous LC3-II or an mCherry-EGFP-LC3B reporter upon lysosomal blockade suggested a modest increase in autophagic flux in TDR HEp-2 cells (Fig 4a and S5 Fig). Next, we measured the autophagic clearance of aggregated p62 in parental and TDR HEp-2 cells by immunofluorescence analysis of p62 accumulation upon distal autophagic inhibition by lysosomal blockade. Besides confirming higher steady-state p62 protein abundance in TDR HEp-2 cells, as already observed in Fig 3, we found remarkably higher accumulation of p62+ puncta upon treatment, revealing higher lysosomal digestion over time of aggregated p62 in TDR HEp-2 cells as compared to parental counterparts (Fig 4b). Demonstrating a causal role in chemoresistance, effective genetic silencing of the essential autophagic gene ATG7 (S6a Fig) restored parental sensitivity to combined drug treatment in TDR HEp-2 cells, while having no effect on drug sensitivity of parental HEp-2 cells (Fig 4c). Similarly, pharmacological inhibition of autophagy with bafilomycin A1 increased resistant cell sensitivity to levels comparable to parental counterparts (Fig 4d).

**Fig 5:**
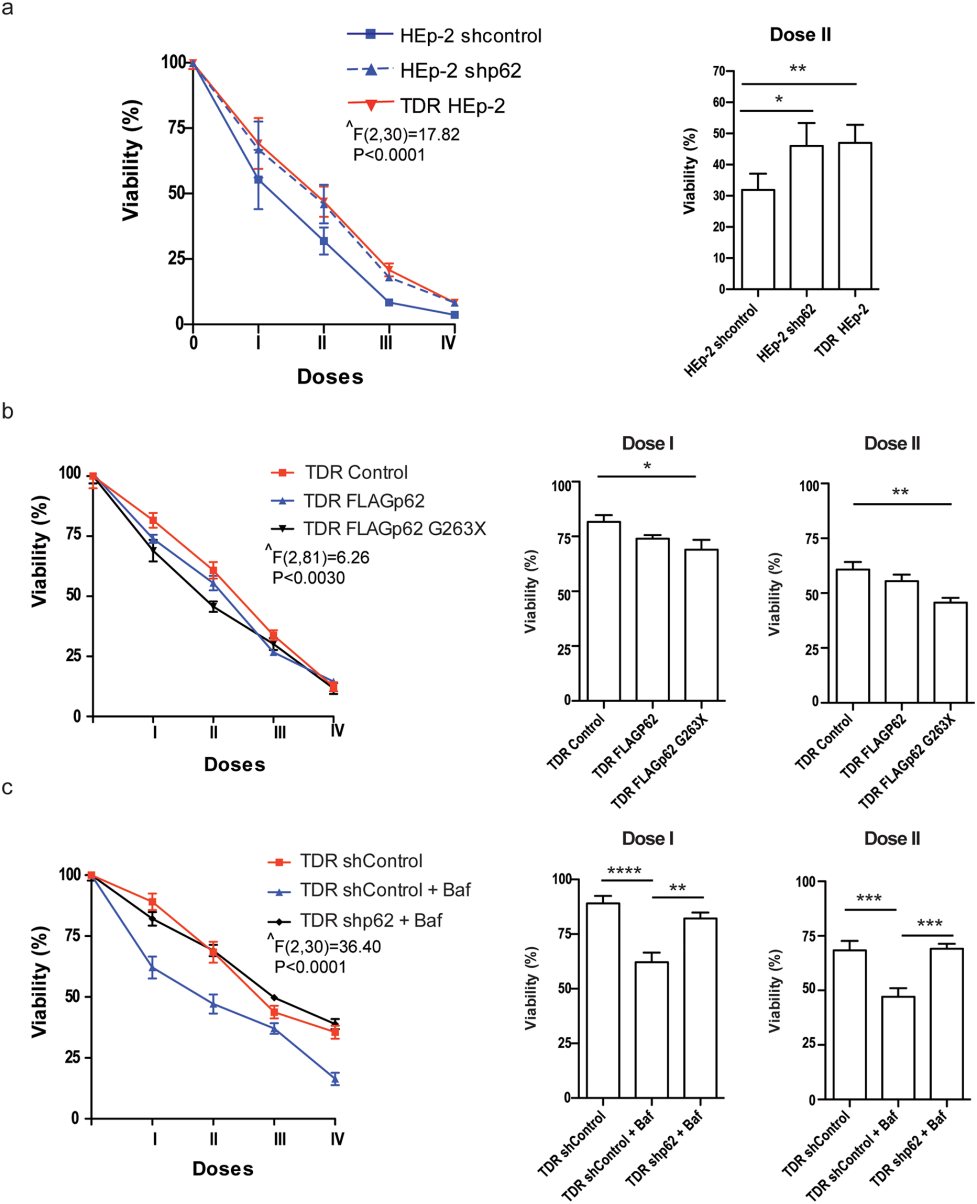
Role of p62 in chemoresistance in HEp-2 cells. (**a**) Cell viability in response to combined treatment with increasing combined doses of cisplatin, 5-FU, and docetaxel for 24 h in stably p62-silenced parental HEp-2 as compared with mock-transduced HEp-2 (shcontrol) and TDR HEp-2 cells, by MTT assay. **I-IV** indicate the progressively doubling concentrations of cisplatin (μM), 5-FU (μM) and docetaxel (nM), respectively: **I**, 1, 20, 3; **II**, 2, 40, 6; **III**, 4, 80, 12; **IV**, 8, 160, 24. (**b**) Effect of combined treatment with increasing doses of cisplatin, 5-FU, and docetaxel on cell viability, as in (a), in TDR HEp-2 cells engineered to express a FLAG epitope-tagged full length p62 (FLAGp62) or a FLAG-tagged truncated mutant lacking both the ubiquitin- and LC3-binding domains (FLAGp62 G263X), as compared with control mock-transduced TDR HEp-2 cells (Control). The western blot analysis of full-length and truncated p62 expression in engineered TDR HEp-2 cells is shown in S6c Fig. (**c**) Assessment of combined drug toxicity as in (a) and (b) in TDR HEp-2 cells treated with bafylomycin-A1 (Baf) upon stable lentiviral p62 silencing (shp62) as compared to mock-transduced TDR HEp-2 cells (shControl). (**a-c**) Left: dose-response viability curves. Right: effect exerted by the indicated combination (mean ± SEM). Two-way ANOVA (group x treatment) with Bonferroni post-hoc test, *p < 0.05, **p < 0.01, *** *p* < 0.001; **** *p* < 0.0001; n = 4 in (**a**), 6 in (**b**), 3 in (**c**). **^** main effect of group.

We then tested the role of p62 in chemosensitivity through genetic manipulation in parental and TDR HEp-2 cells. Silencing efficiency by stable lentiviral expression of a specific short hairpin RNA against p62 (shp62) was ascertained both at the transcript and protein level by qRT-PCR and immunoblotting, the latter showing ~80% reduction of the target protein in shp62-treated cells (S6b and S6c Fig). Ablation of p62 did not alter basal cell viability (not shown), but reduced sensitivity to combined drug treatment in parental HEp-2 cells, which became as resistant as TDR HEp-2 cells (Fig 5a). Conversely, the expression of a truncated p62 mutant lacking both the autophagic domains (the ubiquitin- and LC3-binding domains) and the Nrf2 activating domain (Keap1 interacting region, KIR) significantly enhanced drug sensitivity. This mutant can neither be engulfed in autophagosomes nor activate Nrf2, but its aggregogenicity through the N-terminal PB1 domain is maintained. Notably, the over-expression of wild-type p62 showed only modest effects on chemoresistance, suggesting that specific domains of p62, such as KIR, may also mediate protection against anticancer agents, as previously described [13]. Moreover, ablation of p62 completely prevented autophagic inhibition from increasing chemosensitivity of TDR HEp-2 cells (Fig 5c).

Altogether, the data suggest a key role played in the generated chemoresistance by autophagy, mediated, at least in part, by clearance of toxic p62^+^ aggregates.

## Discussion

Cancer cells are “addicted” not only to oncogenes, but also to genes that mediate essential cytoprotective, stress-adaptive pathways and are not responsible for initiating tumorigenesis. Indeed, stress phenotypes, (*i*.*e*., DNA damage, replicative, oxidative, mitotic, metabolic, and proteotoxic stress) are common features of transformed cells. As a result, cancer cells heavily rely on stress response pathways not only for survival, but also to resist pharmacological treatments, offering valuable therapeutic opportunities [2]. In this study, we generated an *in vitro* cellular model by conditioning the epithelial cancer cell line Hep-2 with increasing doses of three standard chemotherapeutic agents: cisplatin, 5-fluorouracil, and docetaxel, single and combined, resulting in modestly, but consistently reduced drug sensitivity and investigated the underlying molecular bases. We highlighted that autophagy plays an important role in the obtained drug resistance. Indeed, we found basal higher expression of the autophagy receptor p62 and other autophagic genes and higher activity of the Nrf2 anti-oxidant pathway in triple drug resistant (TDR) cells as compared to parental HEp-2 cells (Figs 2 and 3). Higher Nrf2 activity was adaptive, as TDR cells were protected from ROS accumulation upon drug re-administration (Fig 3). Moreover, resistant cells showed slightly higher autophagic activity, and, in particular, higher autophagic clearance of p62^+^ aggregates (Fig 4). Conversely, other stress response pathways, like the ER and mitochondrial UPR, and proteasome subunit expression were not concertedly up-regulated in TDR cells.

Autophagy and Nrf2 have been reported to exert both oncosuppressive and pro-tumoral roles [14, 15]. These pathways constitutively maintain cell homeostasis, by preventing accumulation of toxic ROS and aggregated proteins, as well as DNA damage, thus opposing malignant transformation. Indeed, *Nrf2* knockout mice revealed increased propensity to chemical carcinogen-induced bladder, stomach and skin tumorigenesis [16, 17]. Similarly, mouse strains lacking the essential autophagic genes *ATG5* and *ATG7* develop liver damage, inflammation and benign liver tumors unable to progress to carcinoma [18]. This observation suggests that autophagy may be important to suppress initial malignant transformation in healthy cells, but also essential for tumor progression. Indeed, in established tumors, cytoprotective pathways endowed with tumor suppressor activity are constitutively activated to cope with increased stress. In keeping with this, Nrf2 demonstrated a pro-survival and clonogenic role in cancer cells, accounted for by its role against hypoxia and oxidative stress [19]. Moreover, autophagy was found to be hyperactivated in hypoxic tumor regions where it appeared essential for tumor growth, since knocking down the expression of crucial autophagic genes in tumor cell lines remarkably reduced survival [20,21].

The expression of autophagic genes may also hold prognostic significance. For example, in oral squamous cell carcinoma, autophagic markers were associated with poor overall survival and tumor recurrence [22]. Furthermore, association between Nrf2, autophagy and chemoresistance has been reported in many tumors, including non-small cell lung cancer, stomach cancer, endometrial cancer, and osteosarcoma [23]. Of particular relevance for the present study, O’Donovan and colleagues demonstrated that esophageal cancer cell lines able to induce autophagic gene expression upon cisplatin and 5-FU treatment displayed higher chemoresistance; moreover, specific inhibition of autophagy with siRNAs targeting *BECN1* and *ATG7* transcripts significantly enhanced 5-FU sensitivity [24]. In line with these observations, the pharmacological inhibition of the Nrf2 pathway has recently emerged as a promising approach as adjuvant therapy to enhance chemotherapy efficacy, and several modulators are already available and under investigation [25]. Similarly, many autophagy inhibitors have been tested in preclinical studies in cancer therapy, while others are currently employed in clinical trials [26]. In particular, the therapeutic potential of chloroquine, a drug with documented anti-autophagic activity, long used as an anti-malarial agent, is being increasingly explored in clinical cancer treatment, where it appeared well tolerated, both alone and in combination with other chemotherapeutic agents [27].

Nrf2 is a transcription factor activated by oxidative stress, via the regulated action of the redox-sensitive inhibitory protein Keap1 [8]. Autophagy is a pleiotropic catabolic strategy with many protective functions activated by multiple intracellular and environmental conditions [28]. The ability of p62, the prototypical receptor for selective autophagy, constitutively degraded during the process, to stabilize and activate Nrf2 through the inhibitory binding with Keap1, links these two pathways, implying the possibility of their co-modulation during stress responses, with a putative role in chemotherapy resistance [9]. Moreover, Nrf2 is known to transactivate p62, providing a relevant feedback loop [29]. In our model we found significant induction of p62, along with other Nrf2 targets in TDR HEp-2 cells, and that p62 silencing in these cells resulted in a marked reduction of Nrf2 protein and target gene expression (S4 Fig), suggesting a causal role of the p62/Nrf2 axis in adaptation to chemotherapeutic agents.

Moreover, the observed heightened autophagic clearance of p62+ aggregates in TDR HEp-2 cells (Fig 4) led us to challenge the role of p62 in chemoresistance. Lentiviral p62 silencing suggested a toxic effect of p62 accumulation. Indeed, stable p62 silencing was sufficient to increase chemoresistance in parental HEp-2 cells, suggesting that the inefficiency in maintaining homeostatic levels of p62 through autophagic degradation might play a crucial role in chemotherapy-induced cytotoxicity (Figs 4 and 5). Harboring diverse moieties and controlling different signaling activities, the multifaceted roles of p62 in cancer pathobiology and chemoresistance are not fully elucidated. Recently, two papers from the Karin laboratory showed that p62-dependent Nrf2 activation is essential for the development of hepatocellular carcinoma (HCC) and malignant progression of pancreatic ductal adenocarcinoma [30,31], suggesting a pro-tumoral activity of p62. Notably, the ectopic expression of wild-type or UBA-deleted, but not of a KIR-mutated p62 promoted malignant transformation in HCC mice models [30]. In keeping with this evidence, Saito and colleagues demonstrated that Nrf2-activating phosphorylation of the p62 KIR domain increases chemoresistance, proliferation and malignancy of HCC, confirming the cancer protective role of p62-dependent Nrf2 activation [13].

However, beyond this protective anti-oxidant effect, p62 accumulation may also result in maladaptive cellular stress. Indeed, p62+ protein aggregates have been associated with, and to play a causal role in different diseases. In 2007, Komatsu and colleagues demonstrated marked toxic accumulation of p62 and ubiquitinated proteins in inclusion bodies in *ATG7*-deficient mice [32]. Similar p62-positive inclusions have been identified frequently as protein aggregates in neurodegenerative disorders such as Parkinson and Alzheimer’s diseases, as well as in alcoholic and steato-hepatitis [33]. In these pathologies, the generation of such inclusion bodies may be caused by insufficient autophagic degradative capacity. Indeed, in the *ATG7* knockout model, the loss of p62 was associated with marked reduction of ubiquitin-positive inclusions and oxidative stress, suggesting that aggregation depends on p62 [32] and that p62 aggregation may exacerbate stress in cancer cells [34]. Of note, we reported that proficient p62-mediated autophagic degradation of protein aggregates plays a crucial protective role in the hematological malignancy, multiple myeloma that suffers from constitutive proteotoxic stress, and heavily relies on the UPS and autophagy for survival [21]. Moreover, aggregated p62 has been documented and proposed to have prognostic significance in oral squamous cell carcinoma [35, 36].

In view of these possible roles of p62 in cancer, we asked if the chemoresistance achieved in our model might depend on the higher autophagic activity displayed by resistant cell populations, and in particular on the clearance of toxic p62+ aggregates. Our study documented a key role played by autophagy in the *in vitro* generated chemoresistance of HEp-2 cells, mediated by the efficient management of p62 accumulation. Indeed, pharmacological inhibition of autophagy resulted in p62 accumulation and completely restored parental chemosensitivity of TDR HEp-2 cells (Fig 4). Moreover, p62 ablation prevented autophagy inhibition from restoring parental sensitivity in TDR HEp-2 cells and was sufficient to generate chemoresistance in HEp-2 cells (Fig 5). Finally, forced expression of a double mutant of p62 lacking autophagic and Nrf2-activating domains was sufficient to increase drug-induced toxicity (Fig 5). Altogether, these findings indicate that HEp-2 cells rely on autophagy to survive in drug-induced stress conditions and indicates–at least in our *in vitro* model–that autophagy plays a key role in conferring chemoresistance to carcinoma cells by coping with p62-related proteotoxicity. Our conclusions are consistent with a model of dual roles of p62 in cancer, with a protective anti-oxidant function conferred by the KIR domain [13, 30, 31] and a maladaptive role related to its aggregation and resulting oxidative stress and proteostatic insufficiency [32, 34]. Depending on the cellular context one function of p62 may prevail, highlighting the therapeutic potential of domain-specific inhibitors of p62. Consistently, Saito et al. showed that a KIR-specific inhibitor of p62 specifically hindered Nrf2 activation and increased the sensitivity of HCC to anticancer agents [13]. Conversely, in the context of our cellular model, the maladaptive function of p62 seems to be more relevant, at least in response to the drugs tested. This may also be related to a novel specific function of p62 in inhibiting DNA repair recently proposed as a relevant mechanism of resistance to DNA-damaging agents [37, 38].

In conclusion, our data disclose that the autophagic machinery might confer chemoresistance through increased capacity to manage chemotherapy-induced oxidative and proteotoxic stress. This observation provides a framework to identify potential targets for the therapeutic exploitation of non-oncogene addiction in human epithelial cancers.

## Supporting information

S1 Fig Sensitivity of HEp-2 cells previously conditioned with increasing concentrations of cisplatin, 5-FU, or docetaxel (respectively, Cis HEp-2, 5FU HEp-2 and Doce HEp-2) and of parental HEp-2 cells to 24 h treatment with the indicated drug concentrations, as measured by MTT assay (mean ± SEM, two-way ANOVA with Bonferroni post-hoc test, *p < 0.05; ** *p* < 0.01; *** *p* < 0.001; n = 4). (TIF)

S2 Fig Sensitivity of parental, single drug-conditioned (Cis HEp-2, 5FU HEp-2 and Doce HEp-2) and triple drug resistant (TDR) HEp-2 cells to the indicated 24 h treatments, as assessed by MTT assay. Asterisks indicate statistical significance of differences between the relative cell line and parental HEp-2 cells (mean ± SEM, one-way ANOVA with Bonferroni post-hoc test, *p < 0.05; ** *p* < 0.01; n = 4).
(TIF)

S3 Fig Expression of the indicated transcripts (a) and of p62 protein (b) in parental, single drug-conditioned (Cis HEp-2, 5FU HEp-2 and Doce HEp-2) and triple drug resistant (TDR) HEp-2 cells (mean ± SEM, one-way ANOVA with Bonferroni post-hoc test, *p < 0.05; ** *p* < 0.01; *** *p* < 0.001; n = 3). (TIF)

S4 Fig (a) Expression of p62 and Nrf2 proteins in control or p62 silenced TDR HEp-2 cells treated with cisplatin 4 μM + 5-FU 80 μM + docetaxel 12 nM (three drugs, 3D) for 24 h. (b) Expression of the Nrf2-target mRNA, HMOX1 and NQO1 in p62-silenced TDR HEp-2 cells (mean ± SEM, Welch t-test, *p < 0.05; ** *p* < 0.01; *** *p* < 0.001; n = 3).
(TIF)

S5 Fig (a) Immunofluorescent analysis of autophagic flux in parental and TDR HEp-2 cells transfected with the mCherry-EGFP-LC3B reporter and treated with 10 nM bafilomycin-A1 (Baf) for 16 h. Scale bar, 10 μm. (b) Cytofluorimetric assessment of mCherry-EGFP-LC3B accumulation in parental and TDR HEp-2 cells treated as in (a). Rel. MFI: Median EGFP fluorescence intensity in Baf-treated cells normalized on untreated cells.
(TIF)

S6 Fig **(a)** Effective stable lentiviral silencing of ATG7 at the protein level in HEp-2 cells. (**b-c**) Effective stable lentiviral silencing of p62 at the protein (b) and transcript (c) level in HEp-2 cells. (**d**) Western blot analysis of exogenous expression of FLAG epitope-tagged full length and G263X mutant p62 in TDR HEp-2 cells.
(TIF)

S1 Table Increasing drug concentrations adopted for chemoresistance induction. (DOCX)
