# Supplementary figures and images for: Autophagy mediates epithelial cancer chemoresistance by reducing p62/SQSTM1 accumulation
Source: PLoS One. 2018 Aug 1;13(8):e0201621. doi: 10.1371/journal.pone.0201621 (PMC6070274; doi:10.1371/journal.pone.0201621)

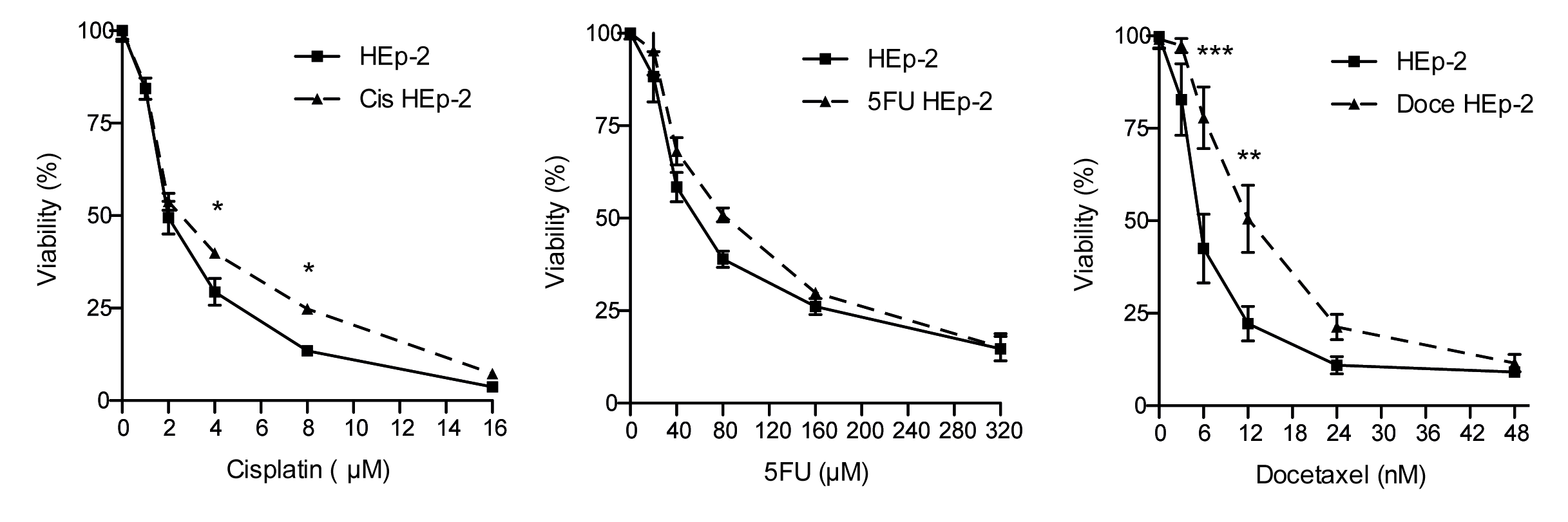

Supplement: S1 Fig — (TIF) [file pone.0201621.s001.tif]

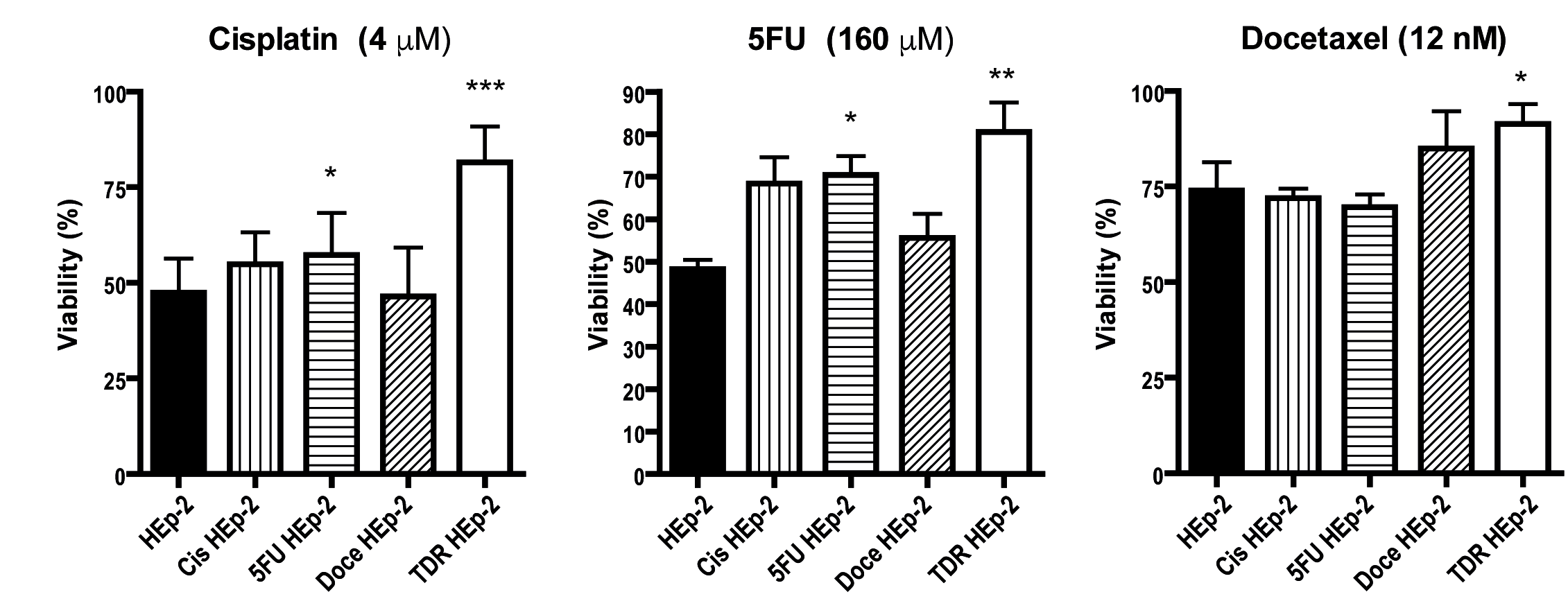

Supplement: S2 Fig — Asterisks indicate statistical significance of differences between the relative cell line and parental HEp-2 cells (mean ± SEM, one-way ANOVA with Bonferroni post-hoc test, *p < 0.05; ** p < 0.01; n = 4). (TIF) [file pone.0201621.s002.tif]

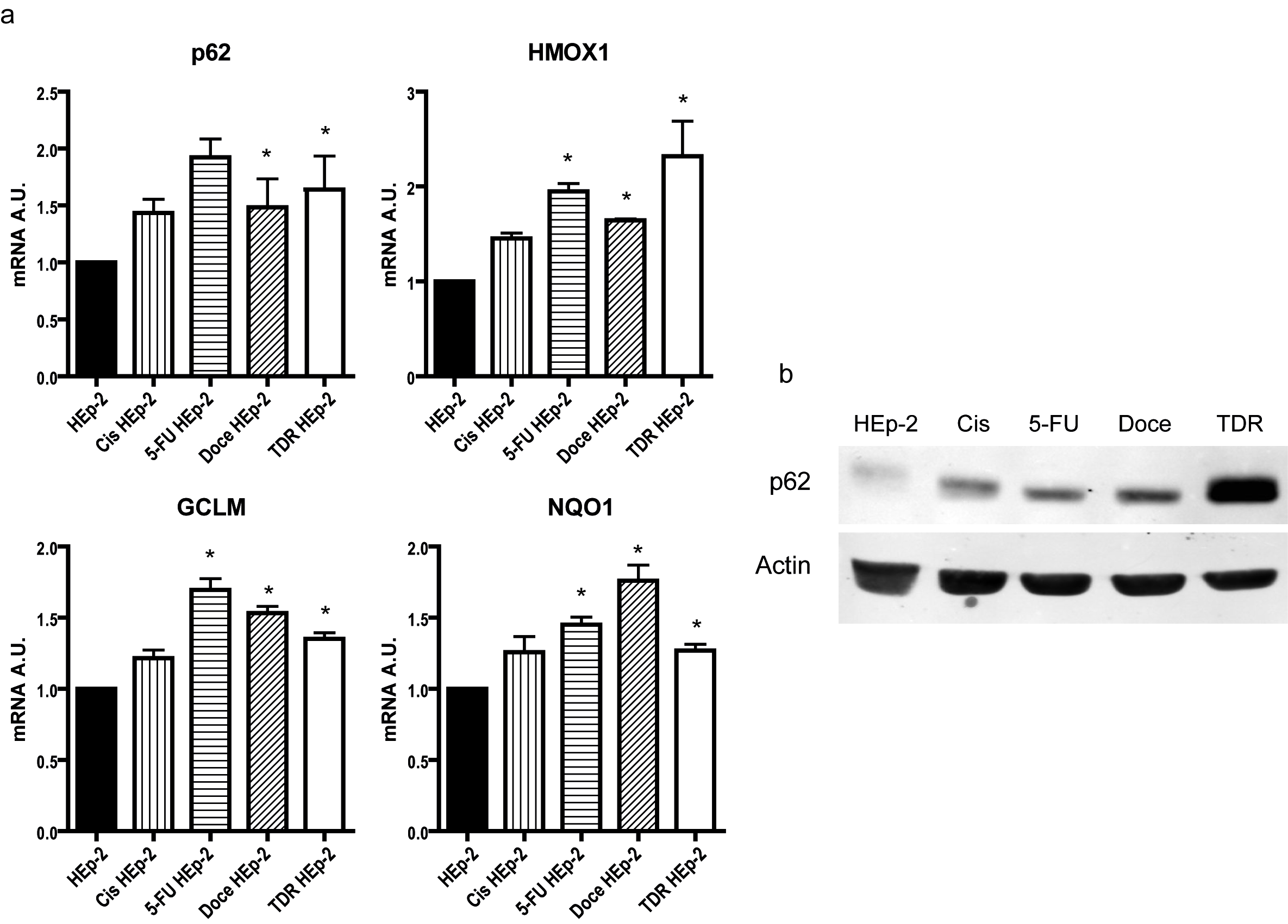

Supplement: S3 Fig — (TIF) [file pone.0201621.s003.tif]

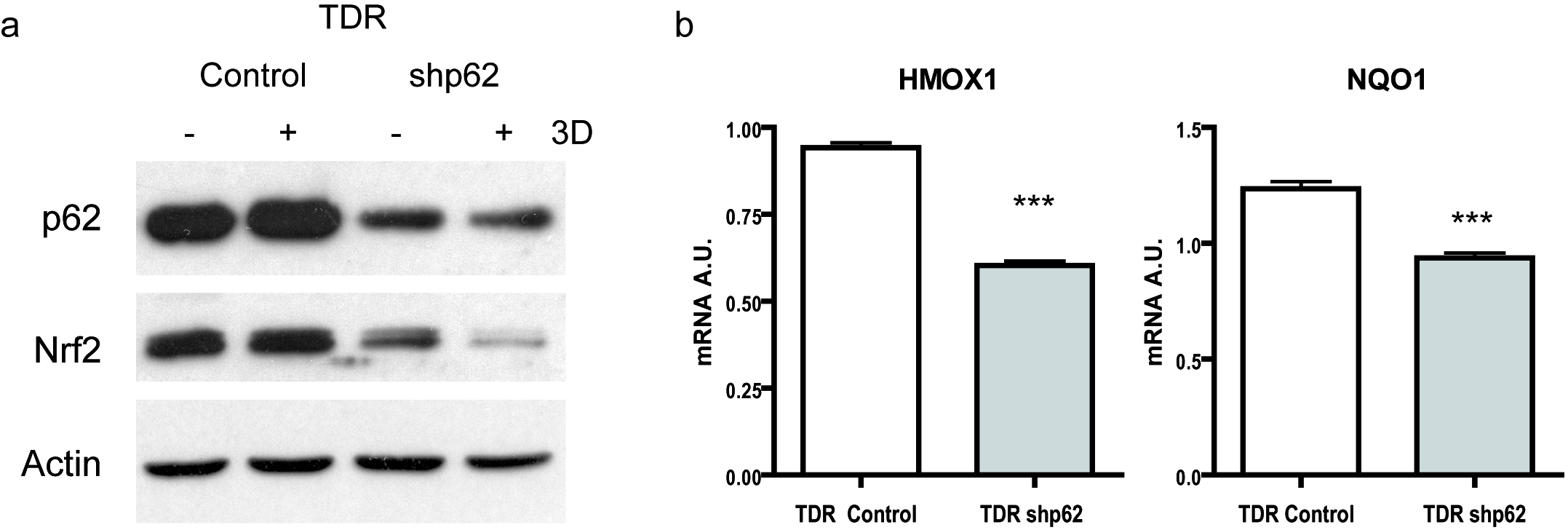

Supplement: S4 Fig — (a) Expression of p62 and Nrf2 proteins in control or p62 silenced TDR HEp-2 cells treated with cisplatin 4 μM + 5-FU 80 μM + docetaxel 12 nM (three drugs, 3D) for 24 h. (b) Expression of the Nrf2-target mRNA, HMOX1 and NQO1 in p62-silenced TDR HEp-2 cells (mean ± SEM, Welch t-test, *p < 0.05; ** p < 0.01; *** p < 0.001; n = 3). (TIF) [file pone.0201621.s004.tif]

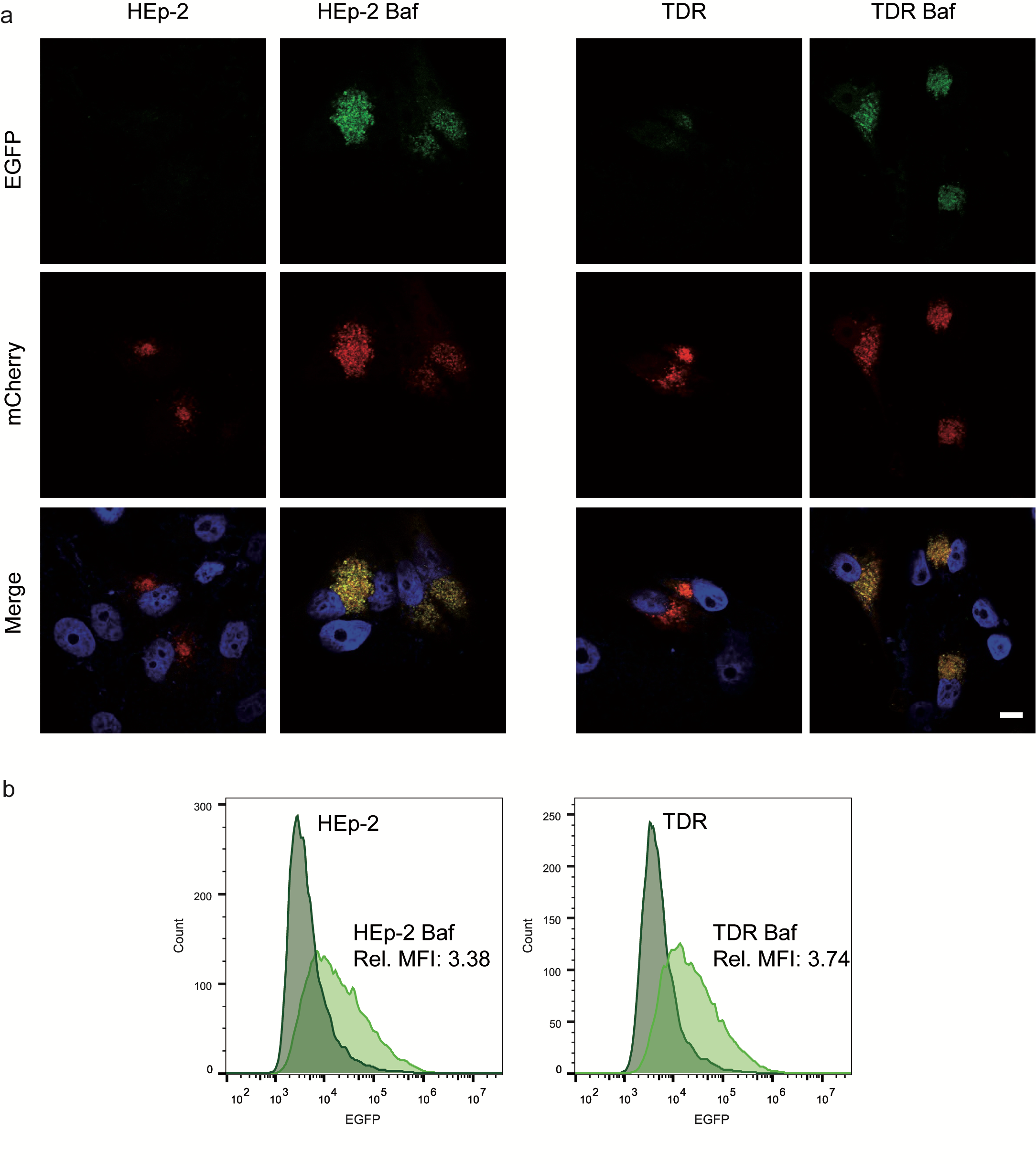

Supplement: S5 Fig — (a) Immunofluorescent analysis of autophagic flux in parental and TDR HEp-2 cells transfected with the mCherry-EGFP-LC3B reporter and treated with 10 nM bafilomycin-A1 (Baf) for 16 h. Scale bar, 10 μm. (b) Cytofluorimetric assessment of mCherry-EGFP-LC3B accumulation in parental and TDR HEp-2 cells treated as in (a). Rel. MFI: Median EGFP fluorescence intensity in Baf-treated cells normalized on untreated cells. (TIF) [file pone.0201621.s005.tif]

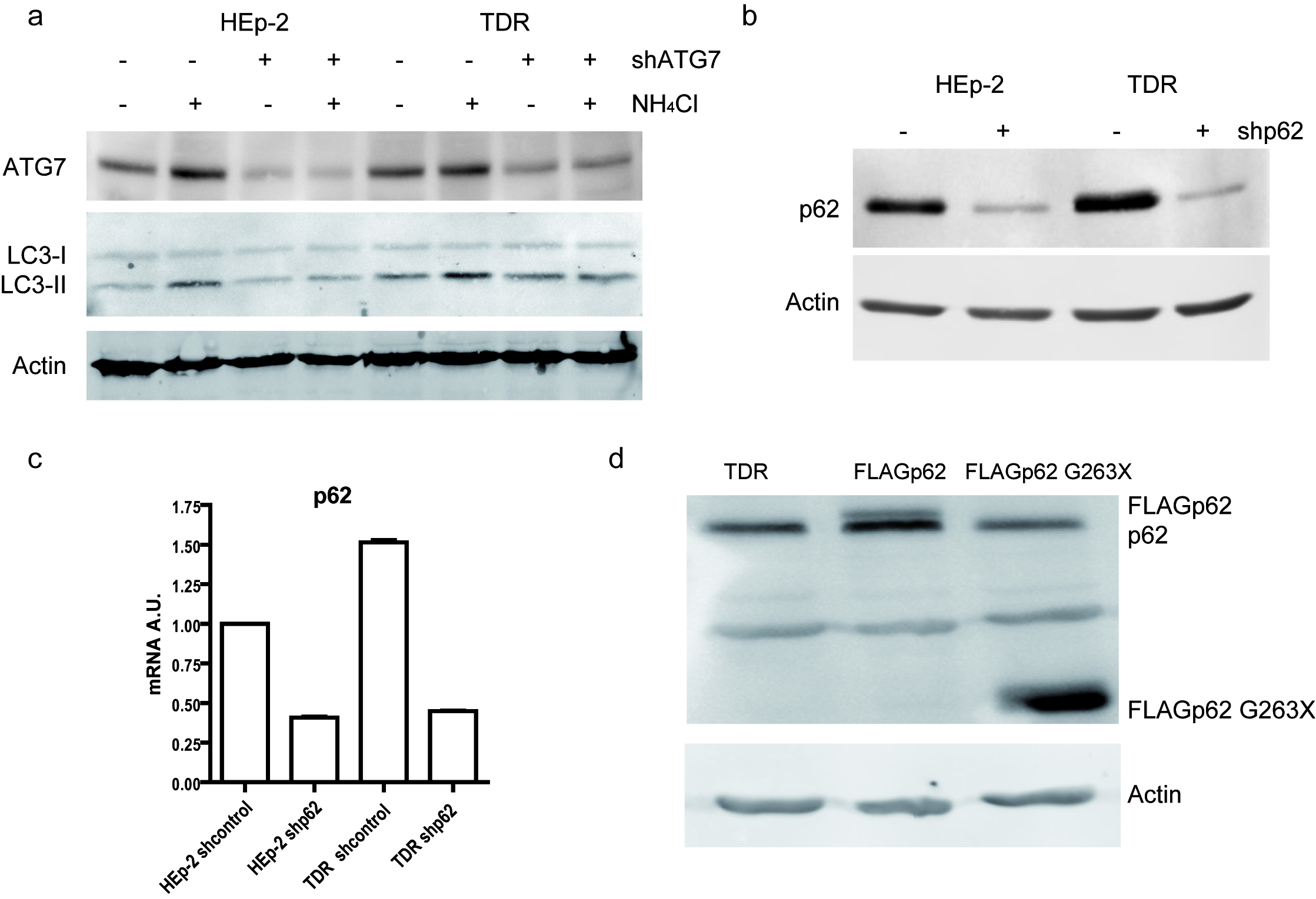

Supplement: S6 Fig — (a) Effective stable lentiviral silencing of ATG7 at the protein level in HEp-2 cells. (b-c) Effective stable lentiviral silencing of p62 at the protein (b) and transcript (c) level in HEp-2 cells. (d) Western blot analysis of exogenous expression of FLAG epitope-tagged full length and G263X mutant p62 in TDR HEp-2 cells. (TIF) [file pone.0201621.s006.tif]
