# Supplementary material for: Autophagy mediates epithelial cancer chemoresistance by reducing p62/SQSTM1 accumulation
Source: PLoS One. 2018 Aug 1;13(8):e0201621. doi: 10.1371/journal.pone.0201621 (PMC6070274; doi:10.1371/journal.pone.0201621)
Supplement: S1 Table — (DOCX) [file pone.0201621.s007.docx]

|  | **Combination dose** | | |
| --- | --- | --- | --- |
| **Drug** | **I** | **II** | **III** |
| Cisplatin (μM)  5-FU (μM)  Docetaxel (nM) | 1 | 2 | 4 |
|  | 20 | 40 | 80 |
|  | 3 | 6 | 12 |
|  |  |  |  |

**S1 Table.** **Increasing drug concentrations adopted for chemoresistance induction.**
